# Cryptic aspergillosis: a rare entity and a diagnostic challenge

**DOI:** 10.1099/acmi.0.000344

**Published:** 2022-04-12

**Authors:** Lavanya R., Marilyn M. Ninan, Regi Kurien, Fouzia N. A, Rani D. Sahni, Joy S. Michael

**Affiliations:** ^1^​ Department of Clinical Microbiology, Christian Medical College, Vellore 632004, India; ^2^​ Department of ENT 3 and Anterior Skull Base Surgery, Christian Medical College, Vellore 632004, India; ^3^​ Department of Hematology, Christian Medical College, Vellore 632004, India

**Keywords:** cryptic species, MALDI-TOF MS (matrix-assisted laser desorption/ionization time-of-flight mass spectrometry), ITS sequencing, AFST (antifungal susceptibility testing), MEC (minimum effective concentration)

## Abstract

**Introduction:**

Cryptic aspergillosis, caused by cryptic species of *Aspergillus*, is increasingly reported in humans and causes significant morbidity and mortality in immunocompromised individuals. The main aim of this study was to describe the occurrence of this entity at a large tertiary care centre and analyse the challenges in identifying them in a routine diagnostic laboratory.

**Methods:**

This was a retrospective case review of all patients diagnosed with cryptic *Aspergillus* species from April 2019 to February 2020. The isolates were identified using conventional microbiological techniques, matrix-assisted laser desorption/ionization time-of-flight mass spectrometry (MALDI- TOF MS), 28S rRNA and internal transcribed spacer (ITS) sequencing.

**Results:**

The species identified were *Aspergillus tamarii, Aspergillus lentulus* and *Aspergillus sydowii*. Identification by MALDI- TOF MS and sequencing was concordant for all except *A. sydowii*, with MALDI- TOF MS misidentifying it as *Aspergillus thermomutans*. All isolates showed low minimum inhibitory concentrations (MICs) for the panel of antifungal drugs.

**Conclusion:**

Aspergillosis caused by cryptic *Aspergillus* species presents a diagnostic challenge. This study confirms the importance of molecular methods for accurate identification.

## Introduction

*Aspergillus* species cause a wide range of infections that may be local or disseminated. Manifestations range from aspergilloma, allergic aspergillosis [1], bronchopulmonary aspergillosis, otomycosis, keratitis, endophthalmitis, cutaneous wound infections and invasive aspergillosis [2, 3].

The International Commission of *Penicillium* and *Aspergillus* retains *Aspergillus* spp. as a single comprehensive genus [4]. The current classification consists of 4 subgenera, namely *Aspergillus, Circumdati*, *Nidulantes* and *Fumigati*, and 20 sections. The most frequently isolated species from clinical samples include *Aspergillus flavus* and *Aspergillus fumigatus*. The genus *Aspergillus* now consists of 339 species [5]. The classification of the genus *Aspergillus* is based on morphological characteristics. For accurate species identification, analyses of morphological, physiological and molecular characteristics are required. Enhanced identification capacities, such as matrix-assisted laser desorption/ionization time-of-flight mass spectrometry (MALDI- TOF MS) and sequencing, have led to the identification of previously unknown ‘cryptic species’ [4]. Cryptic species cannot be diagnosed by morphology but are independent evolutionary lineages in nature that have poorly differentiated morphology and have been misinterpreted as members of single species [6]. Certain striking features, including poor sporulation and low *in vitro* susceptibility to antifungals, led to the discovery of a new fungus, *Aspergillus lentulus*, which was the first cryptic species to be identified. Soon after *A. lentulus,* one more cryptic species, *Aspergillus thermomutans*, was identified within the *Fumigati* section. Coral reefs are affected by an emerging fungal pathogen named *Aspergillus sydowii*, another cryptic species, also known as *Emericella sydowii*, which causes sinusitis, onychomycosis and keratomycosis [7]. The prevalence of cryptic *Aspergillus* species has been poorly investigated. Two studies from the USA and Spain have shown a prevalence of 10 and 15%, respectively, in clinical settings [8]. The main aim of this study was to describe the prevalence of this entity and analyse the different species identified and their susceptibility patterns in a routine diagnostic laboratory.

## Methods

This was a retrospective study undertaken at a tertiary care centre in India from April 2019 to February 2020. Ethical approval was obtained from the Institutional Review Board (IRB MinNo.13344). Forty-three isolates of *Aspergillus* spp. were isolated, of which six could not be morphologically identified on conventional culture methods, including Sabouraud’s dextrose agar with and without antibiotics, as well as brain heart infusion agar (Table S1, available in the online version of this article). Of these, four isolates were retrievable and underwent identification by MALDI-TOF MS version 3.2, confirmed by 28S rRNA and internal transcribed spacer (ITS) sequencing. Antifungal susceptibility testing by broth microdilution (minimum inhibitory concentration, MIC) and E-tests (minimum effective concentration, MEC) were performed for all four isolates against amphotericin B, voriconazole, posaconazole, itraconazole and caspofungin, and the values were interpreted according to the Clinical and Laboratory Standards Institute (CLSI) M38 (third edition, 2017) [9, 10]. Briefly, amphotericin B, voriconazole and itraconazole (Molekula, UK) and posaconazole(Sigma-Aldrich, India) were prepared with Roswell Park Memorial Institute 1640 culture media (RPMI) (Sigma-Aldrich, India) at final concentrations of 0.03–16 µg ml^−1^ in 96-well microtitre plates. The isolate conidial suspensions, adjusted to 0.4–5×10^4^ c.f.u. ml^−1^, were prepared from 48 h cultures on Sabouraud’s dextrose agar. Echinocandin susceptibility was tested by E-test (AB Biodisk, France) with a drug concentration range of 0.002–32 µg ml^−1^. This was performed with an isolate conidial suspension matched to 0.5 McFarland turbidity and plated on RPMI-1640 Glucose MOPS agar prepared as per the manufacturer’s instruction. The MEC was determined at 24 h for echinocandins and the MIC at 48 h for azoles and amphotericin B. The MIC values obtained were classified as wild-type and non-wild-type, based on the CLSI epidemiological cutoff values previously used against these cryptic species [11, 12]. *Candida krusei* ATCC 6258 and *Candida parapsilosis* ATCC 22019 were used as quality controls for each susceptibility test performed and with each batch of E-tests. The quality controls run for all assays were satisfactory.

## Case 1

A 51 year old (Table 1) presented with complaints of facial pain and was diagnosed with chronic invasive fungal sinusitis with intracranial extension and uncontrolled diabetes mellitus and hypertension. Endoscopic debridement and palatectomy was carried out and a biopsy from the right spheno- ethmoidal recess grew *Aspergillus* species. MALDI-TOF MS identified this as *Aspergillus tamarii*, which was confirmed by sequencing. Treatment was begun with intravenous amphotericin B 70 mg once daily for 6 weeks and the patient was well at discharge.

**Table 1.** Case presentation

Case	Sample type	Diagnosis	MALDI-TOF MS	Sequencing	Immune status	Antifungal susceptibility testing	Neutrophil counts	Medical management	Surgical management	Outcome
**1**.	Tissue (right spheno-ethmoidal recess)	Chronic invasive fungal sinusitis	*A. tamarii*	*A. tamarii*	Immunocompetent	Pan-susceptible	76%	I/V amphotericin B	Endoscopic debridement and palatectomy	Well at discharge
**2**.	Tissue (right maxillary sinus)	Allergic fungal rhino sinusitis	*A. tamarii*	*A. tamarii*	Immunocompetent	Pan-susceptible	Not available	No antifungals; oral amoxicillin and prednisolone	Submucosal resection with septoplasty, FESS	Well at discharge
**3**.	Tissue (left lung aspergilloma)	Left lung aspergilloma hyper IgE syndrome	*A. lentulus*	*A. lentulus*	Immunocompromised	Pan-susceptible	66%	I/V amphotericin B, oral voriconazole	Thoracotomy with decortication of aspergilloma	Died
**4**.	Tissue (brain biopsy)	Multiple fungal brain abscess, beta thalassemia major	*A. thermomutans*	*A. sydowii*	Immunocompromised	Pan-susceptible	52%	I/V amphotericin B, oral voriconazole, posaconazole	Right parietal craniotomy, excision of abscess	Died

I/V, Intravenous.

## Case 2

A 32 year old (Table 1) with a known history of recurrent allergic fungal sinusitis presented with complaints of bilateral nasal block, more on the right, associated with mucoid nasal discharge, paroxysmal sneezing and intermittent bilateral frontal heaviness. Bilateral full functional endoscopic sinus surgery (FESS) was carried out. Tissue sample from the right maxillary sinus grew *Aspergillus* spp. and MALDI-TOF MS identified this as *A. tamarii*, which was confirmed by sequencing. Amoxicillin 500 mg thrice daily for a week and prednisolone 50 mg once daily for 10 days were given. No antifungals were given and recovery was uneventful.

## Case 3

A 9-year-old child (Table 1), a known case of primary immunodeficiency with hyper IgE syndrome and a DOCK 8 mutation, presented to the paediatric outpatient with complaints of fever and difficulty in breathing for 20 days. The patient history revealed an apparently normal child at birth, who developed generalized maculopapular rash at day 9 of life and was diagnosed to have pneumonia, skin and renal abscesses, and pyelonephritis subsequently. Immunization was as per schedule up to 5 years of age. A chest radiograph showed a left lung pleural effusion and a mediastinal mass. Initial treatment was with intravenous meropenem, vancomycin and clindamycin. Ultrasound-guided biopsy of the lung mass was carried out and the fungal culture of the biopsy grew *Aspergillus* spp., which was speciated by MALDI- TOF MS as *A. lentulus* and confirmed by sequencing. Treatment was with intravenous amphotericin B and oral voriconazole was added on the microbiological diagnosis of the same. A thoracotomy with decortication was carried out, and despite these measures the child succumbed to the illness 4 days later.

## Case 4

A 14 year old child (Table 1) who was a known case of beta thalassemia presented with a history of right-sided weakness and facial deviation for a week and generalized tonic clonic seizures a day before hospitalization. The child was on regular treatment and underwent a matched unrelated bone marrow transplant at a previous admission. At this admission, a CT brain showed multiple ill-defined hypodense lesions and an MRI brain scan showed multiple brain abscesses in the right frontal cerebellum and bilateral parietal and thalamic regions. The biopsy grew *Aspergillus* species. MALDI-TOF MS identified this as *A. thermomutans,* but sequencing identified *A. sydowii*. Right parietal craniotomy and excision of abscess were carried out and the patient was initially started on liposomal amphotericin B and voriconazole but changed to posaconazole following susceptibility testing. The patient gradually developed hypotension, spiralled into sepsis and expired a month later.

## Discussion

Published literature suggests that the occurrence of cryptic species is of importance in stem cell/solid organ transplant patients and its clinical spectrum includes invasive aspergillosis, pneumonia, exacerbation of chronic obstructive pulmonary disease (COPD), keratitis, eyelid infection, onychomycosis, cutaneous infections, wound infection, invasive nasal aspergillosis and nasal polyposis [13, 14]. Unfortunately, there are no clinical data for guiding the management of aspergillosis caused by cryptic species. Azoles and amphotericin B are the agents of choice to treat *Aspergillus* infections and early initiation of an effective antifungal therapy has a direct impact on patient survival [15]. However, due to the low frequency of cryptic *Aspergillus* species in clinical practice, they are not likely to inﬂuence the choice of empirical or primary antifungals. The minimum inhibitory concentration (MIC) for azoles is usually high for cryptic *Aspergillus* species, while amphotericin B commonly retains activity [4].

The case review of the patients diagnosed with cryptic aspergillosis provides insights into their rarity, invasiveness, different species, and methods of identification and treatment. In our series, we have isolated *Aspergillus tamarii* from two immunocompetent individuals, as well as *Aspergillus lentulus* and *Aspergillus sydowii* from immunocompromised children (Table 1). In contrast to the published literature describing cryptic isolates with poor sporulation, the isolates in the current study sporulated after 3 days of incubation, with the exception of *A.sydowii*, which did not sporulate. Identification by MALDI-TOF MS and sequencing was concordant, except for *A. sydowii*, which was misidentified as *A. thermomutans* by MALDI-TOF MS, and in addition had decreased antifungal susceptibility. Again, in contrast to the published literature describing cryptic isolates with decreased antifungal susceptibility, all of our isolates (Table 2) displayed low MICs for the panel of antifungal drugs tested [11]. Despite the *in vitro* susceptibility of *A. lentulus* and *A. sydowii* to all antifungals, the two immunocompromised patients in this study succumbed to their illnesses.

**Table 2.** Antifungal susceptibility testing results

Isolate MIC (µg ml^−1^)	Amphotericin B	Voriconazole	Itraconazole	Posaconazole	Caspofungin E-test
***A. tamarii***	1	0.5	0.25	0.06	0.064
***A. tamarii***	0.5	0.25	0.25	0.06	0.042
***A. lentulus***	0.25	0.25	0.25	0.03	Not available
***A. sydowii***	0.25	2	0.5	0.25	0.125

MIC, Minimum Inhibitory Concentration.

The common *Aspergillus* species isolated from clinical samples in India include *A. lentulus, A. thermomutatus, A. udagawae, A. viridinutans, A. fumigatiaffinis, A. novofumigatus* in the *Fumigati* section, *A. alliaceus* in the *Flavi* section, *A. carneus* and *A. alabamensis* in the *Terrei* section, *A. tubingensis* and *A. awamori* in the *Nigri* section, *A. sydowii* in the *Versicolores* section, *A. westerdijkiae* and *A. persii* in the *Circumdati* section, and *A. calidoustus, A. insuetus* and *A. keveii* in the *Usti* section (Table 3). MALDI-TOF MS was used to identify these rare species and antifungal susceptibility testing (AFST) showed that most of the species were susceptible to posaconazole and echinocandins [16]. In Korea the most common *Aspergillus* species include *A. awamori* and *A. tubengensis*, followed by *A. sydowii*, *A. lentulus* and *A. tamarii*. In Brazil, *Aspergillus* species belonging to the section *Flavi* – mainly *A. tamarii* – were most commonly reported from clinical specimens [17].

**Table 3.** List of cryptic species and their sections adapted from Nedel *et al*. [18]

Aspergillus section	Cryptic species
***Fumigati***	***A. lentulus*** *A. thermomutans* *A. udagawae* *A. viridinutans* *A. fumigatiaffini* *A. novofumigatus*
***Flavi***	***A. tamarii*** *A. alliceus*
***Terrei***	*A. carneu* *A. alabamensis*
***Nigri***	*A. acidus* ***A. tubingensis*** *A. awamori*
***Versicolores***	***A. sydowii*** *A. creber*
***Circumdatii***	*A. persii* *A. westerdijkia*
***Usti***	*A. calidoustus* *A. insuetus* *A. keveii*

Since cryptic *Aspergillus* species cause invasive disease and decreased drug susceptibilities [18], correct identification and characterization of these fungi may have both epidemiological and clinical significance. With a widening immunocompromised population in India and an increasing incidence of invasive fungal infections, there is a need for rapid and accurate identification of unidentified *Aspergillus* at species level along with knowledge of their epidemiological cutoff values. Diagnostics such as sequencing have a positive impact and can inform antifungal treatment strategies and therefore play a crucial role in dealing with rare species. Larger datasets are needed for correlation with clinical disease and to establish trends [19].

## Supplementary Data

Supplementary material 1
