# Supplementary material for: Cryptic aspergillosis: a rare entity and a diagnostic challenge
Source: Access Microbiol. 2022 Apr 12;4(4):000344. doi: 10.1099/acmi.0.000344 (PMC9260091; doi:10.1099/acmi.0.000344)
Supplement: Supplementary material 1 [file acmi-4-0344-s001.pdf]

Supplementary Table 1: Diagnostic methodology

Case	No. of samples received at this admission	Cultures plated *per sample (n=5)	Grown *Aspergil lus spp*	Histopathology report	Radiological evidence	Other samples received and organism grown if any
**1**	1	SAB(2)<br>SDA(2)<br>BHIA(1)	**4/5**	**Chronic invasive fungal rhinosinusitis** and evidence of acute invasive fungal sinusitis, **fungal elements are seen.**	None	None
**2**	1	SAB(2)<br>SDA(2)<br>BHIA(1)	**4/5**	**Chronic rhinosinusitis**, benign inflammatory polyp and allergic mucin with **septate fungal hyphae resembling *Aspergillus* spp**	CT Paranasal sinuses: Expansion of paranasal sinuses with soft tissue opacification and hyper density in the sinuses, **fungal infection to be ruled out**	**Filamentous fungi**
**3**	1	SAB(2)<br>SDA(2)<br>BHIA(1)	**4/5**	Decortication specimen from left lung tissue-Acute invasive sinusitis, **septate and occasional** aseptate fungal hyphae.	CT Thorax: **Necrotic components** of lung mass and pleural effusion	**Filamentous fungi**
**4**	1	SAB(2)<br>SDA(2)<br>BHIA(1)	**4/5**	Fungal meningoencephalitis with abscess **and evidence of angioinvasion probably due to aspergillus. Fungal hyphae are septate and branching suggestive of *Aspergillus* spp.**	MRI Brain-Multiple ring enhancing lesions with moderate perilesional edema and diffusely scattered in the brain parenchyma-**fungal etiology needs**	**Filamentous fungi**

		**consideration**

*SAB(Sabouraud Dextrose Agar with antibiotics) SDA(Sabouraud dextrose agar without antibiotics) BHIA(Brain heart Infusion Agar)
